# *ECERIFERUM 10* Encoding an Enoyl-CoA Reductase Plays a Crucial Role in Osmotolerance and Cuticular Wax Loading in Arabidopsis

**DOI:** 10.3389/fpls.2022.898317

**Published:** 2022-06-23

**Authors:** Norika Fukuda, Yoshimi Oshima, Hirotaka Ariga, Takuma Kajino, Takashi Koyama, Yukio Yaguchi, Keisuke Tanaka, Izumi Yotsui, Yoichi Sakata, Teruaki Taji

**Affiliations:** ^1^Department of Bioscience, Tokyo University of Agriculture, Tokyo, Japan; ^2^Bioproduction Research Institute, National Institute of Advanced Industrial Science and Technology (AIST), Tsukuba, Japan; ^3^Plant Resources Unit, Research Center of Genetic Resources, NARO, Ibaraki, Japan; ^4^Electron Microscope Center, Tokyo University of Agriculture, Tokyo, Japan; ^5^Nodai Genome Center, Tokyo University of Agriculture, Tokyo, Japan

**Keywords:** osmotolerance, cuticular wax, enoyl-CoA reductase, very-long-chain fatty acid, ER stress, *Arabidopsis thaliana* accession

## Abstract

Acquired osmotolerance induced after salt stress is widespread across *Arabidopsis thaliana* (Arabidopsis) accessions (e.g., Bu-5). However, it remains unclear how this osmotolerance is established. Here, we isolated a mutant showing an acquired osmotolerance-defective phenotype (*aod2*) from an ion-beam-mutagenized M2 population of Bu-5. *aod2* was impaired not only in acquired osmotolerance but also in osmo-shock, salt-shock, and long-term heat tolerances compared with Bu-5, and it displayed abnormal morphology, including small, wrinkled leaves, and zigzag-shaped stems. Genetic analyses of *aod2* revealed that a 439-kbp region of chromosome 4 was translocated to chromosome 3 at the causal locus for the osmosensitive phenotype. The causal gene of the *aod2* phenotype was identical to *ECERIFERUM 10* (*CER10*), which encodes an enoyl-coenzyme A reductase that is involved in the elongation reactions of very-long-chain fatty acids (VLCFAs) for subsequent derivatization into cuticular waxes, storage lipids, and sphingolipids. The major components of the cuticular wax were accumulated in response to osmotic stress in both Bu-5 WT and *aod2*. However, less fatty acids, primary alcohols, and aldehydes with chain length ≥ C30 were accumulated in *aod2*. In addition, *aod2* exhibited a dramatic reduction in the number of epicuticular wax crystals on its stems. Endoplasmic reticulum stress mediated by *bZIP60* was increased in *aod2* under osmotic stress. The only *cer10* showed the most pronounced loss of epidermal cuticular wax and most osmosensitive phenotype among four Col-0-background cuticular wax-related mutants. Together, the present findings suggest that *CER10/AOD2* plays a crucial role in Arabidopsis osmotolerance through VLCFA metabolism involved in cuticular wax formation and endocytic membrane trafficking.

## Introduction

The plant cuticle is an extracellular lipid structure that covers the outer surface of land plants and protects the plant body from abiotic and biotic stresses (Javelle et al., 2011). Cuticle biosynthesis begins in the early stages of embryogenesis and is strictly coordinated with plant growth to supply constant cuticle deposition (Delude et al., 2016). The cuticle is divided into two domains: the underlying cuticular layer, which is a cutin-rich domain with embedded polysaccharides, and the overlying cuticle proper, which is enriched in waxes. A cutin matrix runs through both the cuticular layer and the cuticle proper. Cuticular waxes can be either embedded within the cutin matrix as intracuticular wax or accumulated on the cuticle surface as epicuticular wax crystals or films (Yeats and Rose, 2013; Ingram and Nawrath, 2017). Cuticular wax is a mixture of mostly aliphatic very-long-chain fatty acid (VLCFA; C20–34) derivatives that include alkanes, aldehydes, primary and secondary alcohols, ketones, and esters (Yeats and Rose, 2013). The parent VLCFAs are produced by the activities of four endoplasmic reticulum (ER)-bound enzymes that together form a fatty acid elongase (FAE) complex, and the biosynthesis occurs *via* the following four successive reactions that together form one reaction cycle: (i) condensation of C18-CoA and malonyl-CoA to form 3-ketoacyl-CoA (mediated by 3-ketoacyl-CoA synthase; KCS); (ii) reduction of 3-ketoacyl-CoA to 3-hydroxy-CoA (mediated by ketoacyl-CoA reductase; KCR); (iii) dehydration of 3-hydroxyacyl-CoA to enoyl acyl-CoA (mediated by 3-hydroxyacyl-CoA dehydratase; HCD); and (iv) reduction of enoyl acyl-CoA to form a saturated fatty acyl-CoA product (mediated by enoyl-CoA reductase; ECR). The reaction cycle synthesizes a two-carbon longer acyl-CoA and can be repeated to yield VLCFAs with various chain lengths ranging from C20 up to C38 or more (Batsale et al., 2021). These VLCFAs are then derivatized and subsequently incorporated into the cuticle as cuticular waxes, or converted to suberin, another plant surface barrier on the root endodermis, to triacylglycerol storage lipids, or to membrane lipids such as phospholipids or sphingolipids (Kim et al., 2013; Batsale et al., 2021).

In *A. thaliana* (Arabidopsis), several studies have identified genes encoding the enzymes that form the FAE complex. There are 20 KCS-encoding genes in the Arabidopsis genome, among which *ECERIFERUM 6* (*CER6*)/*KCS6*/*CUTICULAR 1* (*CUT1*) has been shown to play an important role in wax biosynthesis; a *cer6/kcs6/cut1* mutant exhibits a severe waxless phenotype characterized by a decrease in longer wax compounds and accumulation of C24 and C26 derivatives (Millar et al., 1999; Fiebig et al., 2000). Complementation assays using yeast mutants have identified *3-KETOACYL-COA SYNTHASE 1* (*KCS1*) and *PASTICCINO2* (*PAS2*) as Arabidopsis genes encoding a functional KCR enzyme and a functional HCD enzyme, respectively; complete loss of KCR1 or PAS2 activity leads to embryonic lethality, indicating that VLCFA biosynthesis is essential for embryonic development (Bach et al., 2008; Beaudoin et al., 2009). Similar complementation assays using yeast mutants have identified *ECERIFERUM 10* (*CER10*) as an Arabidopsis gene that encodes a functional ECR enzyme (Kohlwein et al., 2001). An Arabidopsis *cer10* mutant is reported to show morphological abnormalities such as reduced size of aerial organs as well as a reduction of cuticular wax load and altered VLCFA composition of seed triacylglycerols and sphingolipids; in addition, the Golgi apparatus is larger and tends to form ring-like clusters, resulting in a possible defect in endocytic membrane transport (Zheng et al., 2005).

Arabidopsis plants subjected to drought, salt, or abscisic acid treatments exhibit a significant increase in the amounts of cuticular lipids in their leaves (Kosma et al., 2009). An R2R3-type MYB transcription factor, *AtMYB49*, has been shown to contribute to salt tolerance in Arabidopsis by increasing cutin deposition in the leaves (Zhang et al., 2020). Ectopic expression of *Newhall* navel orange *CsKCS6*, an ortholog of Arabidopsis *KCS6*, has been shown to increase the amount of VLCFAs in the cuticular wax on the stems and leaves of Arabidopsis plants and to improve drought and salt tolerances (Guo et al., 2020). These reports suggest a relationship between cuticular wax and drought or salt tolerance in Arabidopsis; however, studies are needed to clarify the relationship between FAE complex including ECR and abiotic stress tolerances, and between reduced cuticular wax levels and stress tolerances.

Natural genetic variation in Arabidopsis has facilitated identification of plant genes governing complex traits such as growth, flowering, and stress tolerance (Alonso-Blanco et al., 2009; Weigel, 2012). Previously, we found a wide variation in salt tolerance among Arabidopsis accessions; in addition, we found that while most salt-tolerant accessions also exhibited acquired osmotolerance after salt stress (e.g., Bu-5), others did not (e.g., Col-0; Katori et al., 2010; Ariga et al., 2017); however, little is known about which genes contribute to such acquired osmotolerance. Here, to understand more about the mechanism underlying acquired osmotolerance in Arabidopsis, we isolated and characterized a mutant showing an acquired osmotolerance-defective phenotype (*aod2*) from the Arabidopsis accession Bu-5.

## Materials and Methods

### Plant Materials and Growth Conditions

Arabidopsis seeds (Bu-5 or Col-0) were sown on agar (1%, w/v) plates containing full-strength Murashige and Skoog (MS) medium containing a vitamin mixture (10 mg L^−1^ of myoinositol, 200 μg L^−1^ of glycine, 50 μg L^−1^ of nicotinic acid, 50 μg L^−1^ of pyridoxine hydrochloride, and 10 μg L^−1^ of thiamine hydrochloride; pH 5.7) and 1% sucrose. The plates were sealed with surgical tape and the seeds were stratified at 4°C for 4–7 days before being transferred to a growth chamber (80 μmol photons m^2^ s^−1^; 16 h/8 h light/dark cycle; 22°C) for germination and growth.

To obtain Arabidopsis mutants, Bu-5 seeds were irradiated in the azimuthally varying field cyclotron at the Japan Atomic Energy Agency (Takasaki, Japan). To select the appropriate dose, we irradiated the seeds with carbon ion beams in a dose range of 25–250 Gy and assessed plant development. Doses of 200 Gy or higher inhibited secondary leaf development or induced sterility. Therefore, we irradiated the seeds at 150 Gy in a single layer within a plastic bag.

Seeds of the following four Arabidopsis mutants were obtained from the Arabidopsis Biological Resource Center (Ohio State University): *cer10* (SALK-088645), *lcr* (SALK-136833C), *cer2* (SALK-127158), and *cer5* (SALK-036776).

### Stress Treatment for the Acquired Osmotolerance Assay

Seven-day-old seedlings grown on nylon mesh (990 μm) on an M&S agar plate were transferred with the underlying mesh (hereafter “mesh-transferred”) to a plate supplemented with 100 mM NaCl for 7 days. The seedlings were then mesh-transferred to a plate supplemented with 750 mM sorbitol for 15 days.

### Abiotic Stress Assays

Ten-day-old seedlings grown on nylon mesh (990 μm) on an M&S agar plate were mesh-transferred to a plate supplemented with 200 mM NaCl for 8 days (salt-shock stress), 600 mM sorbitol for 21 days (osmo-shock stress, or 10 μm paraquat for 7 days (oxidative stress). For the long-term heat tolerance assay, the plates with 10-day-old seedlings were placed at 37°C for 4 days and then moved back to 22°C for 5 days. Chlorophyll content was determined as in (Porra et al., 1989).

### RNA Extraction and Quantitative Real-Time Polymerase Chain Reaction

Total RNA extraction and quantitative real-time polymerase chain reaction (qRT-PCR) analysis were performed as described in Isono et al. (2020). *ACTIN2* was used as the internal standard for qRT-PCR. The primers used are listed in Supplementary Table S1.

### Genetic Mapping of the Causative Gene of the *aod2* Phenotype

*aod2* was crossed with an osmo-tolerant accession, Pog-0, and the resulting F_1_ progeny were selfed to generate F_2_ populations. Genomic DNA was prepared from individual F_2_ plants with the recessive phenotype for use as PCR templates. The simple sequence length polymorphism markers listed in Supplementary Table S2 were used for mapping. The PCR conditions were as follows: (94°C for 2 min) 1 cycle, (94°C for 20 s, 52°C–55°C for 20 s, and 72°C for 20 s) 34 cycles, and (72°C for 2 min) 1 cycle. Microsatellites were fractionated in a 5%–7% agarose gel, and the recombination frequencies (%) were calculated from the band pattern.

### DNA Library Construction and Sequencing of *aod2* Mutant

DNA library construction and sequencing were performed as described in (Tsukimoto et al., 2021). The read data were submitted to the DNA Data Bank of Japan Read Archive (accession number DRA013622).

### Detection of Mutations in the *aod2* Mutant

Detection of mutations from the whole-genome sequencing data of the *aod2* mutant was performed as described in Tsukimoto et al. (2021). For detection of the 439-kbp insertion from Chr. 4 into the causal region in the *aod2* mutant, we designed the primer sets listed in Supplementary Table S3. Primer sets (i) and (ii) were designed to detect DNA bands only in the presence of the 439-kbp insertion, and primer set (iii) was designed to detect DNA bands only in the presence of deletion.

### Plasmid Construction and Transformation

For complementation analysis, the genomic region of *AOD2/CER10* (2.0-kb upstream of the ATG initiation codon and 1.0-kb downstream of the termination codon of Bu-5) was amplified by PCR using the pRI909 CER10 primers (Supplementary Table S3) and then cloned into the binary vector pRI909. The constructs were introduced into *Agrobacterium tumefaciens* GV3101, which was used for plant transformation by the floral dip method. Transgenic plants were selected on M&S agar plates containing 200 μg ml^−1^ Claforan and 25 μg ml^−1^ kanamycin. Ten-day-old seedlings (T1 plants) were transferred to soil in pots.

### Toluidine Blue Test

The aerial parts of 2-week-old seedlings were submerged in aqueous solution of 0.05% (w/v) toluidine blue (TB; Sigma, St Louis, MO, United States). After 20 min on a shaker set at 100 rpm, the TB solution was removed, and the plates were washed gently with water to remove excess TB from the plants. Next, the plants were homogenized in a 1.5-ml tube containing zirconia beads. Then, 200 μl of buffer [200 mM Tris–HCl (pH 8.0), 250 mM NaCl, and 25 mM EDTA] and 400 μl of ethanol was added, with vortex mixing, and plant debris was pelleted by centrifugation (15,000 rpm for 10 min). The supernatant was examined spectrophotometrically, and the amount of TB was determined from the absorbance at 630 nm (A630). The major peak of absorbance due to plant material (A435) was used for normalization. Relative levels of TB were calculated as the ratio of A630 to A435 (Tanaka et al., 2007).

### Scanning Electron Microscopy

A 3-cm section from the base of the flower stem 2 weeks after bolting was used for observation of the epidermal surface. The stem was cut into 5–10 mm pieces and coated with Pt + Pd using an E102 ion sputtering system (Hitachi, Tokyo, Japan) and used for scanning electron microscopy (S4800, Hitachi).

### Water Loss Assay

Leaves of 4-week-old plants grown in soil under normal growth conditions were detached and then left in ambient conditions. The weight was measured every 10 min for 1 h. The percentage decreases in fresh weight are expressed as percentage water loss.

### Extraction of Cuticular Waxes and Gas Chromatography-Mass Spectrometry Analysis

Two-week-old Bu-5, *aod2*, and Col-0 seedlings grown on MS agar plates (control condition) and salt-acclimated for 1 week were mesh-transferred to MS agar plates containing 750 mM sorbitol for 21 days (osmo-stress condition). The cuticular wax of leaves (six biological replicates) was extracted by immersion in chloroform containing tricosanoic acid as an internal standard for 10 s. The solvent was evaporated in a steam of nitrogen. Free hydroxyl and carboxyl groups were silylated with N,O-bis(trimethylsilyl)trifluoroacetamide (BSTFA+TMCS, Sigma-Aldrich, St. Louis, MO, United States) for 1 h at 80°C. The wax composition was analyzed by using a GC2020 gas chromatograph (Shimadzu Inc., Kyoto, Japan) with the injector in splitless mode and the following temperature program: increase to 80°C, 15°C per min to 200°C, 3°C per min to 300°C and hold for 10 min at 300°C. Mass spectrum data were obtained on a GCMS-QP2020NX mass spectrometer (Shimadzu) after impact ionization. The peaks were quantified by using the LabSolutions software (Shimadzu). The amount of each wax monomer was determined on the basis of the internal standard and was normalized by leaf surface area. Leaf surface area was calculated by using the ImageJ software (Schneider et al., 2012).

## Results

### Isolation of *aod2*

**Figure 1:**
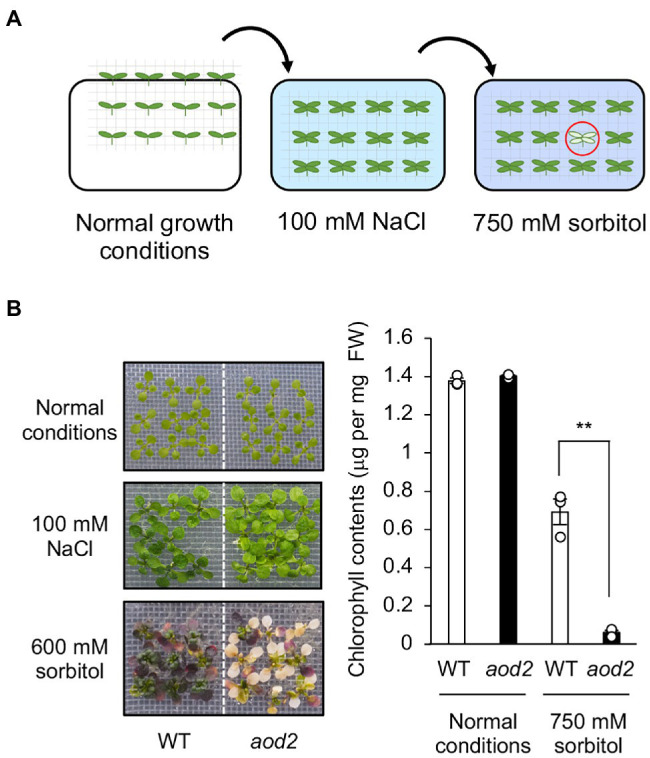
Identification of *acquired osmotolerance-defective* (*aod2*) mutant. **(A)** Flow chart of the acquired osmotolerance assay. A total of 30,000 ion-beam-mutagenized, salt-acclimated, 2-week-old seedlings of accession Bu-5 were transferred to Murashige and Skoog agar plates containing 750 mM sorbitol for 15 days. Seedlings showing osmo-hypersensitivity (red circle) were selected as mutants showing an acquired osmotolerance-defective phenotype. The mutant *aod2* was identified by using this approach. **(B)** Chlorophyll content as an index of acquired osmotolerance in Bu-5 (WT) and *aod2*. FW, fresh weight. Differences between WT (white bar) and *aod2* (black bar) were analyzed by Student’s *t*-test (mean ± SE, *n* = 3, **p < 0.01).

**Figure 2:**
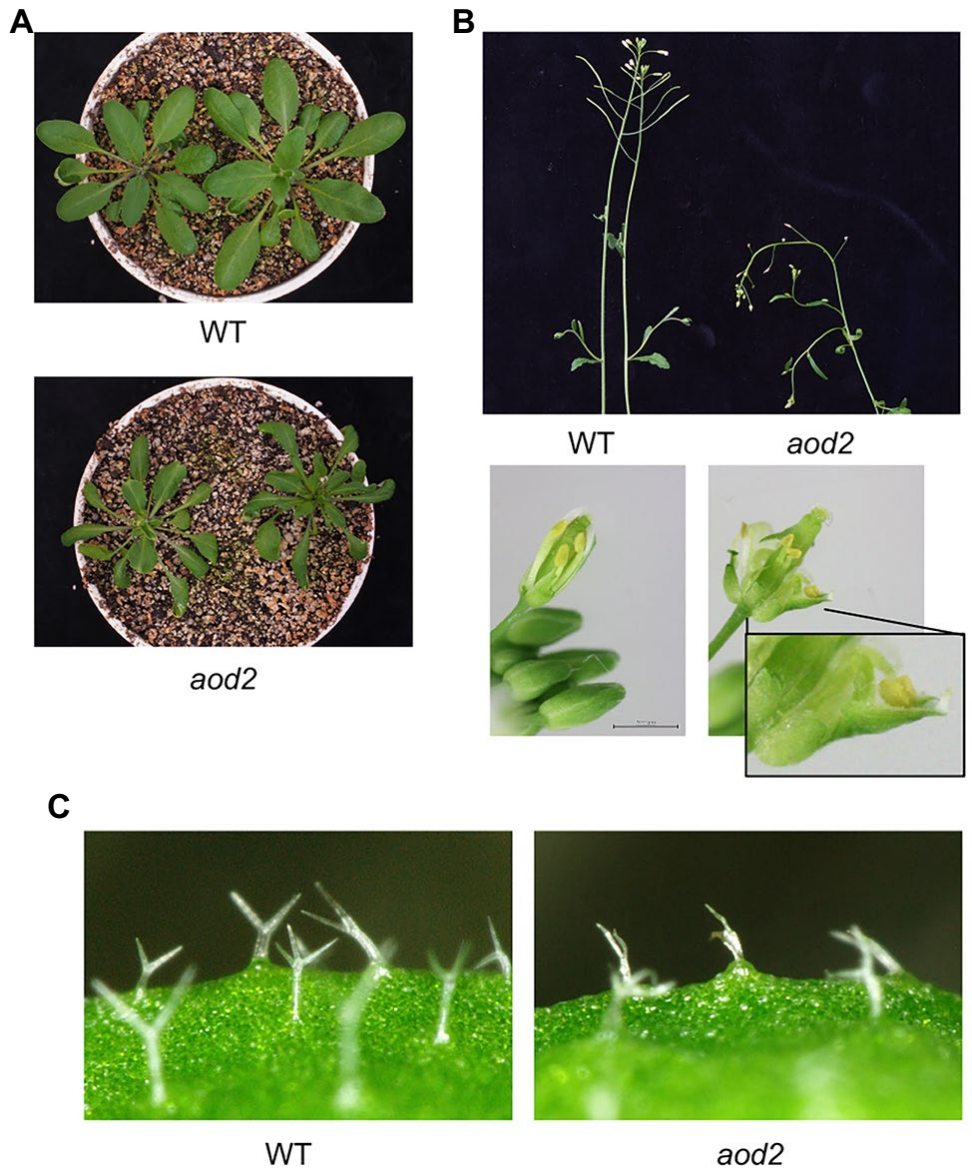
Morphogenesis of *aod2* mutant. Representative images of 4-week-old WT and *aod2* plants grown in soil under normal growth conditions; the images show leaves **(A)**, stems and mechanically opened flowers **(B)**, and leaf trichomes **(C)**.

Previously, we found that Arabidopsis accession Bu-5 showed acquired osmotolerance (Katori et al., 2010; Ariga et al., 2017). Using this accession, we screened 30,000 ion-beam-mutagenized seedlings for mutants showing an acquired osmotolerance-defective phenotype and subsequently obtained the mutant *aod2*. Compared with Bu-5, *aod2* showed significantly lower osmotolerance to 750 mM sorbitol after pre-exposure to 100 mM NaCl for 7 days (Figures 1A,B). In addition, soil-grown *aod2* plants displayed abnormal morphogenesis in all aerial parts compared with Bu-5 plants grown under the same conditions: the leaves of *aod2* plants were narrower and smaller (Figure 2A); the flower stems were shorter and exhibited a zigzag-shaped appearance (Figure 2B); some stamens displayed a zigzag-shaped appearance (Figure 2B); and the leaf trichomes were shorter and crooked (Figure 2C).

### Characterization of *aod2*

**Figure 3:**
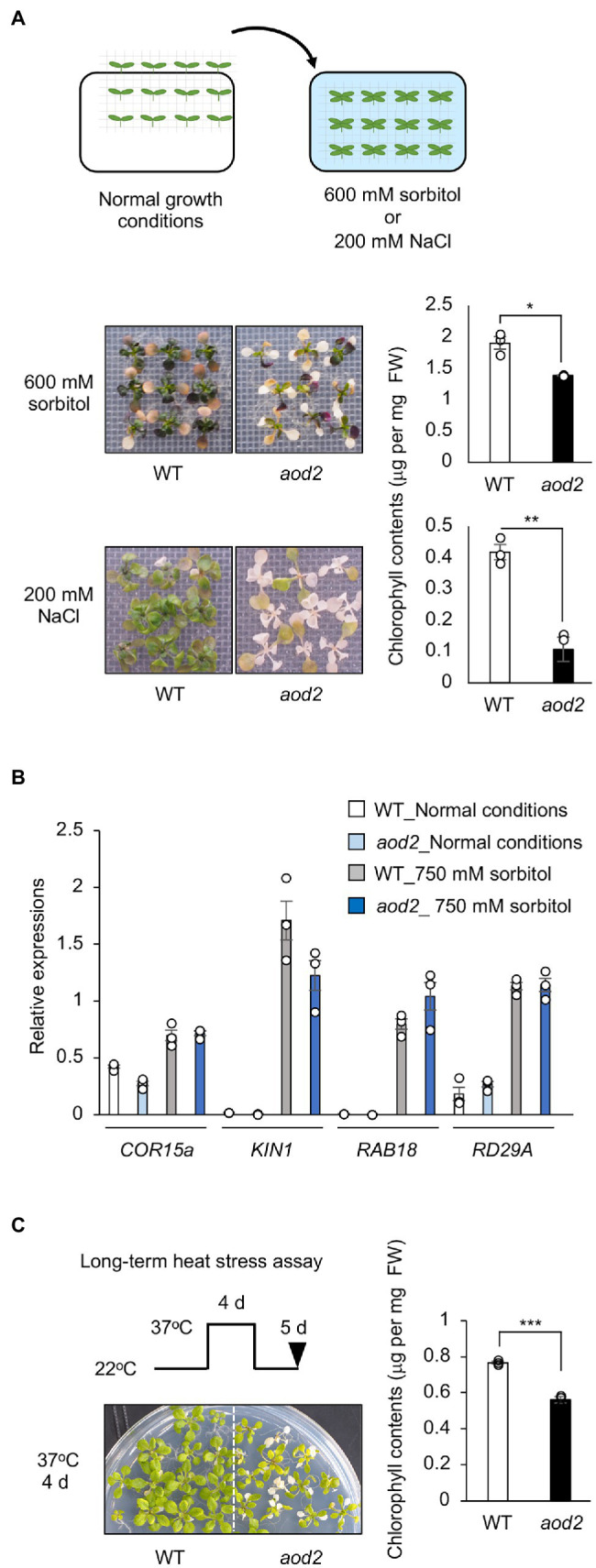
Characterization of *acquired osmotolerance-defective* (*aod2*) mutant. **(A)** Top: flow chart of the salt- and osmo-shock tolerance assay. Two-week-old seedlings were transferred to Murashige and Skoog agar plates containing 200 mM NaCl for 8 days or 600 mM sorbitol for 21 days. Middle and bottom: Chlorophyll content as an index of the salt- and osmo-shock tolerances of *aod2* and Bu-5 (WT). FW, fresh weight. **(B)** Expression of osmostress-responsive marker genes in WT and *aod2* under normal (control) and acquired osmotic stress (100 mM NaCl for 7 days and subsequent 750 mM sorbitol for 8 h) conditions; expression levels were determined by quantitative real-time polymerase chain reaction relative to those of *Actin2* (mean ± SE, *n* = 3). **(C)** Long-term heat tolerance of *aod2*. Ten-day-old WT and *aod2* seedlings were grown initially at 22°C, then at 37°C for 4 days, and then at 22°C for 5 days. Then, chlorophyll content was determined as an index of heat tolerance. Differences between WT and *aod2* were analyzed by Student’s *t*-test (mean ± SE, *n* = 3, **p < 0.01).

To investigate whether the phenotype of *aod2* was specific to acquired osmotolerance, we evaluated the tolerance of *aod2* to three other abiotic stresses—osmo-shock, salt-shock, and oxidative stress (paraquat). *aod2* showed significantly lower osmo-shock and salt-shock tolerance (Figure 3A) but the same oxidative stress tolerance (Supplementary Figure S1) compared with Bu-5. When we compared the expression of the osmostress-responsive marker genes *COR15A*, *KIN1*, *RAB18*, and *RD29A*, we found that the mRNA accumulation levels of all four genes were comparable between Bu-5 and *aod2* (Figure 3B), indicating that the weakened osmotic stress tolerance in the mutant was not due to defective transcriptional regulation. We also found that *aod2* showed significantly greater sensitivity to long-term heat stress (37°C, 4 days) compared with Bu-5 (Figure 3C).

### Identification of the Causal Gene of the *aod2* Phenotype

**Figure 4:**
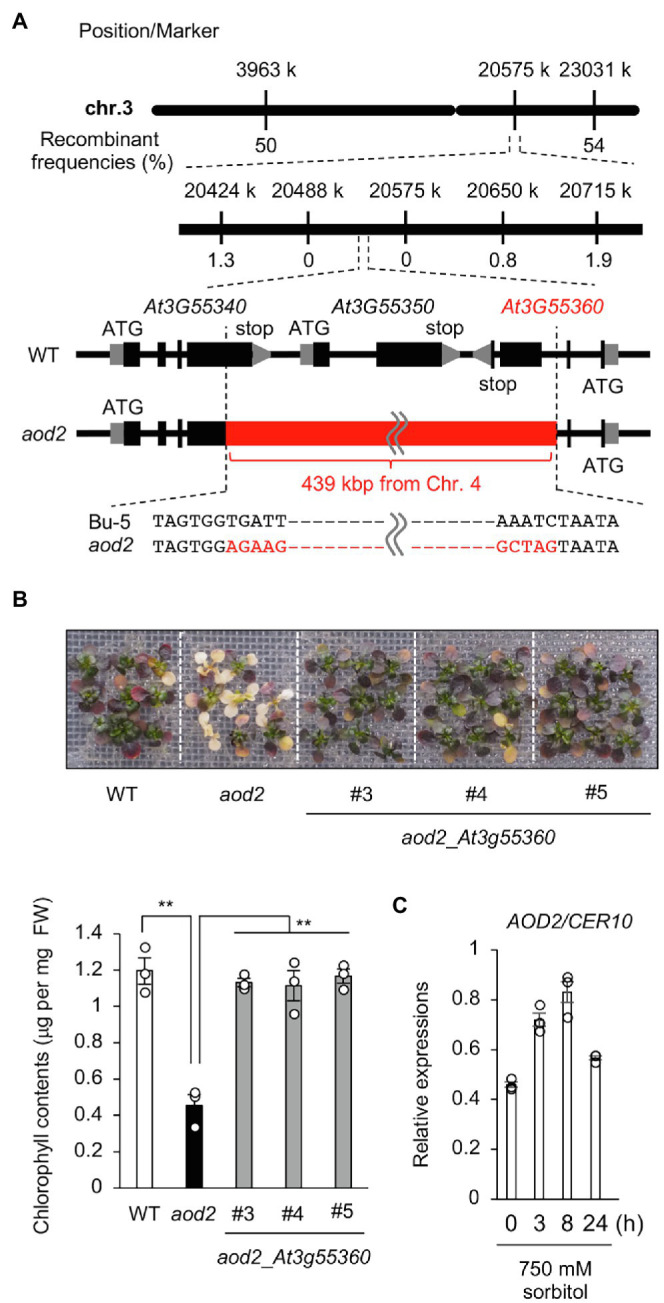
Identification of the causal gene of the acquired osmotolerance-defective phenotype. **(A)** High-resolution mapping of the causal locus in *aod2* by using F_2_ progeny from a cross between *aod2* and Pog-0. The scores indicate recombination frequencies (%). *Arabidopsis* Genome Initiative numbers are shown above the genes. The red bar shows the 439-kbp insertion from Chr. 4. **(B)** Complementation test performed by transforming *aod2* with *AOD2/At3g55360*. T_2_ plants transformed with the native promoter *At3g55360* (*aod2*_*At3g55360*) derived from Bu-5 (WT) were used. Top panel: Representative images showing acquired osmotolerance in the complementation lines. Lower panel: Chlorophyll content of WT, *aod2*, and *aod2*_*At3g55360*. Differences between WT and *aod2* or between *aod2* and *aod2_At3g55360* were analyzed by Student’s *t*-test (mean ± SE, *n* = 3, **p < 0.01). **(C)** Expression profiles of *CER10* in WT under normal and acquired osmotic stress conditions; expression levels were determined by quantitative real-time PCR relative to those of *Actin2* (mean ± SE, *n* = 3). Differences between normal and acquired osmotic stress conditions were analyzed by Student’s *t*-test. **p < 0.01.

To identify the locus responsible for the osmosensitive phenotype of *aod2*, we crossed *aod2* with Pog-0, an accession with acquired osmotolerance (Ariga et al., 2017), and subjected the mutant to high-resolution chromosomal mapping analysis of the locus within a 226-kbp region on the short arm of chromosome 3 (Figure 4A). The sequence variations within this region in the *aod2* genome were examined by whole-genome sequencing. There were no non-synonymous mutations in any of the genes within the 226-kbp region; however, there was a 439-kbp insertion whose sequence was identical to that of part of chromosome 4. To verify the insertion, we performed PCR-based genotyping using primers spanning chromosomes 3 and 4 and confirmed that the 439-kbp insertion disrupted genes *At3G55340*, *At3G55350*, and *At3G55360* (Supplementary Figure S2). To identify which of the disrupted genes was the causal gene of the osmosensitive phenotype *aod2*, we performed complementation tests in which each of the three disrupted genes derived from Bu-5, with their regulatory region, was introduced into *aod2*. Only the introduction of *At3G55360* (*aod2*_*At3G55360*) restored acquired osmotolerance as in Bu-5, indicating that *At3G55360* was the causal gene of the osmosensitive *aod2* phenotype (Figure 4B). *At3G55360* was identical to *CER10*, which encodes an ECR involved in the final stage of the VLCFA elongation reaction cycle that produces VLCFAs for subsequent derivatization to cuticular waxes, storage lipids, and sphingolipids. In Bu-5, the transcription level of *CER10/AOD2* was increased by osmotic stress (Figure 4C).

### Content and Composition of the Cuticular Wax

**Figure 5:**
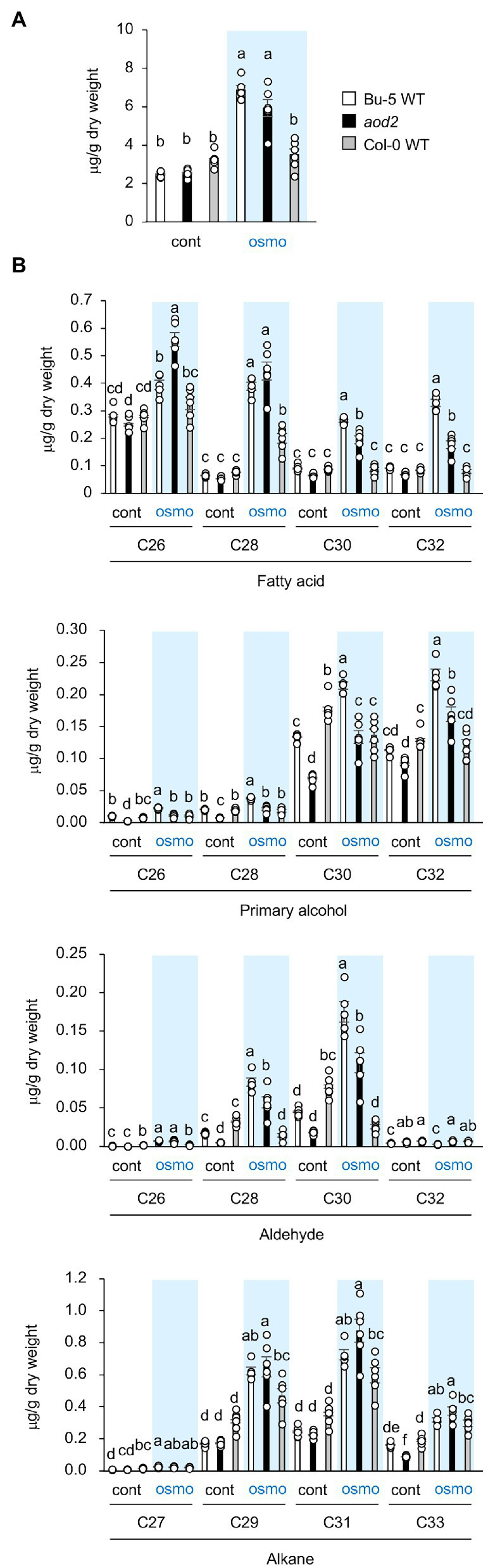
Cuticular wax content and composition in *acquired osmotolerance-defective* (*aod2*) mutant. **(A)** Total wax content (including fatty acids, primary alcohols, aldehydes, and alkanes) in Bu-5, *aod2*, and Col-0 seedlings under normal (cont) and osmotic stress (osmo) conditions. **(B)** Waxes identified in Bu-5, *aod2*, and Col-0 seedlings under normal (cont) and osmotic stress (osmo) conditions. Data are presented as mean ± SE, *n* = 6. Letters at the top of columns are grouped with each chain length based on one-way ANOVA and Tukey’s test, *p* < 0.05.

ECR is an enzyme that catalyzes the final step of VLCFA elongation, and VLCFAs are the precursors of all the aliphatic components of cuticular wax. Therefore, to investigate the biochemical basis for the observed morphological phenotypes in *aod2*, we examined the wax components in the leaves of Bu-5, *aod2*, and Col-0 (a salt-sensitive accession that does not exhibit acquired osmotolerance). First, we examined total wax content (composite of fatty acids, primary alcohols, aldehydes, and alkanes) and found no differences between Bu-5 and *aod2* under control or osmotic stress condition, while Col-0 had less wax content compared to Bu-5 and *aod2* under osmotic stress condition (Figure 5A). When we stratified the wax components into fatty acids, primary alcohols, aldehydes, and alkanes, increases in each type were detected in both Bu-5 and *aod2* under osmotic stress compared with control (Figure 5B). Stratifying by chain length, the amount of each wax component with chain length ≥ C28 in Col-0 was lower than that in Bu-5 under osmotic stress (Figure 5B). In addition, *aod2* had lower amounts of fatty acids, primary alcohols, and aldehydes with chain length ≥ C30 compared with those in Bu-5. The amounts of C26 and C28 fatty acids were higher in *aod2* than in Bu-5, although those of C28 primary alcohols and aldehydes were lower in *aod2* than in Bu-5. The *aod2* exhibited a decrease in C26–C32 fatty acids but an increase in C16 and C18 fatty acids compared to WT (Supplementary Figure S3). No significant difference in alkane content was observed between Bu-5, *aod2*, and Col-0. Together, these findings suggest that accumulation of cuticular wax components plays an important role in the Arabidopsis response to osmotic stress, and that CER10/AOD2 contributes to the biosynthesis of ≥C26–C30 fatty acids, primary alcohols, and aldehydes.

### Wax Layer of *aod2*

**Figure 6:**
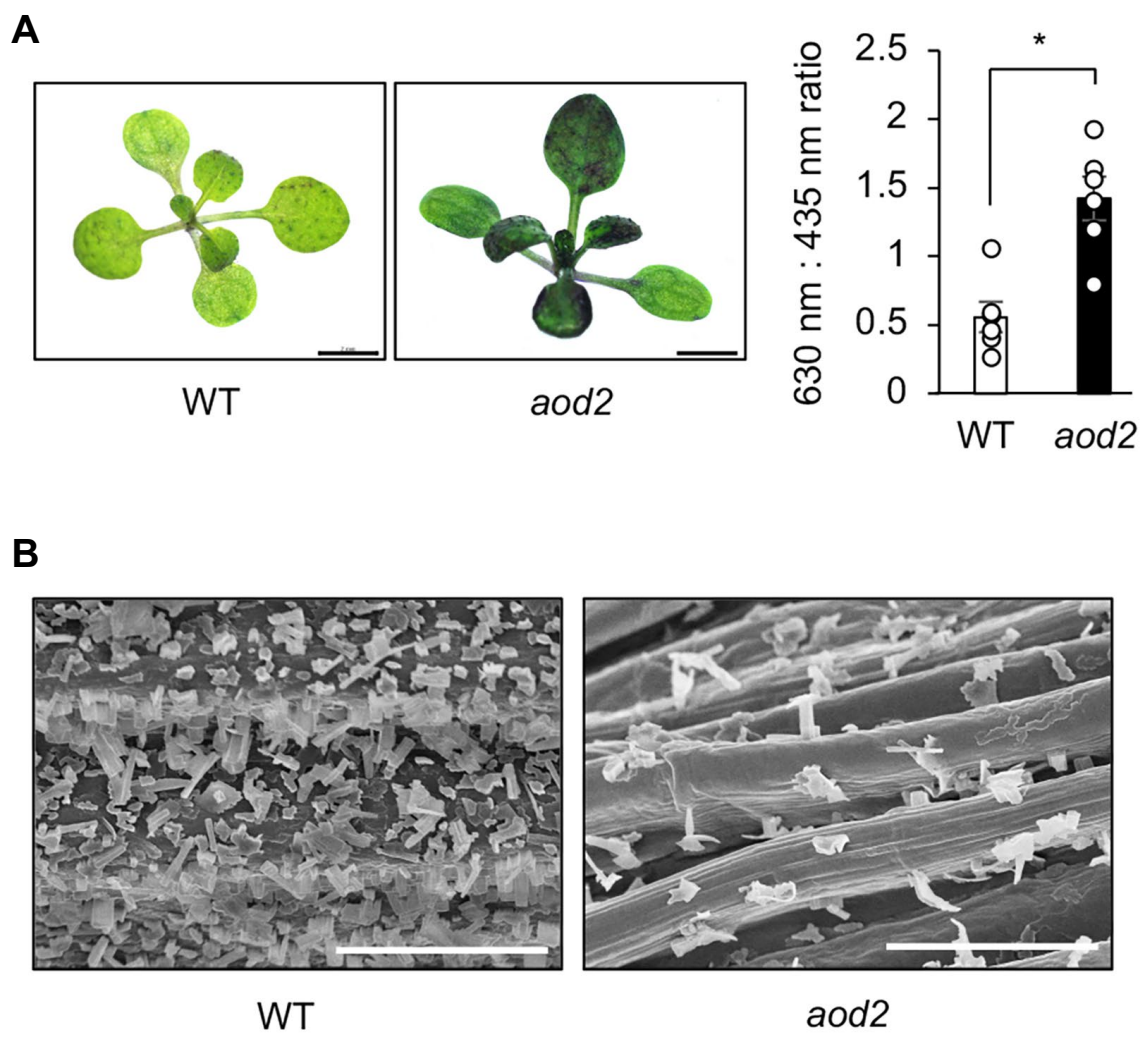
Wax layer of *acquired osmotolerance-defective* (*aod2*) mutant. **(A)** Left: representative images from the toluidine blue (TB) test. Plants with a normal cuticle repel TB, but a deficient cuticle allows TB to permeate the epidermal surface. Two-week-old Bu-5 (WT) and *aod2* plants were used for the experiment. Right: TB uptake was examined spectrophotometrically by measuring absorbance at 630 nm (A630). The major peak of absorbance due to plant material (A435) was used for normalization. Relative levels of TB were calculated as the ratio of A630 to A435. **(B)** Scanning electron microscopy images of the surface of the stems of WT and *aod2*. Bars = 100 μm.

Absence of ECR activity results in a reduction of cuticular wax load in Arabidopsis (Zheng et al., 2005). We therefore examined the integrity of the surface wax layer of the cuticle in *aod2* by using the toluidine-blue (TB) test, which was established for detection of cuticular defects in whole leaf; a deficient cuticle allows TB to permeate the epidermal surface (Tanaka et al., 2004). The true leaves of *aod2* were significantly stained with TB, whereas those of Bu-5 were not (Figure 6A). To investigate the morphology of the surface wax layer, we observed the epicuticular wax crystals on the stems of Bu-5 and *aod2* by scanning electron microscopy; there were many granular wax crystals on the surface of the Bu-5 stems, but much less on the *aod2* stems (Figure 6B). The *aod2*_*At3G55360* plants exhibited wax crystals on the surface of stems like Bu-5 WT plants (Supplementary Figure S4). These findings suggest that CER10/AOD2 plays an important role in the development of epicuticular wax crystals. Although there are differences in the wax contents on the leaf surface as shown in Figure 5, there were no clear differences in leaf surface between WT and *aod2* (Supplementary Figure S5).

### Water Loss From Detached *aod2* Leaves

**Figure 7:**
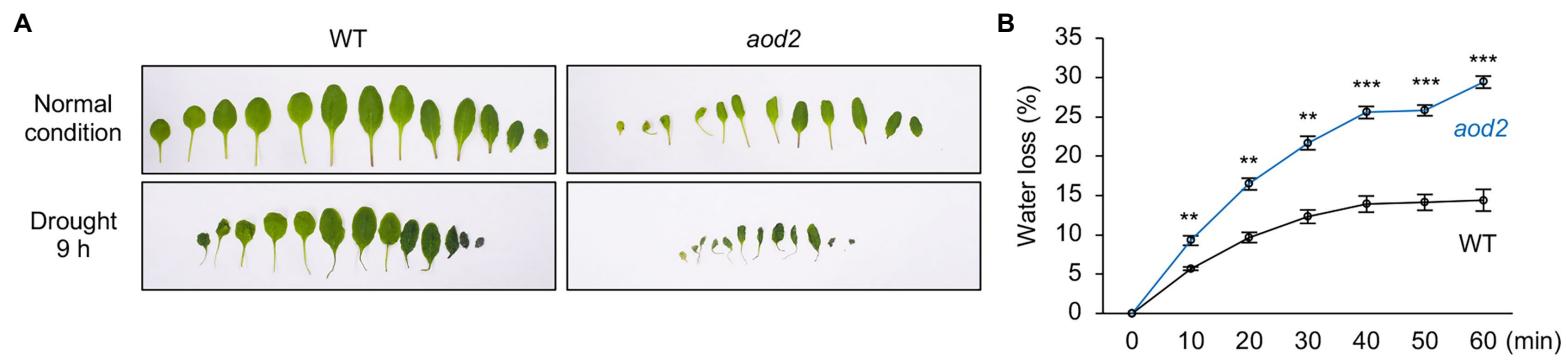
Water loss from detached *acquired osmotolerance-defective* (*aod2*) mutant leaves. **(A)** Leaves of 4-week-old plants grown in soil under normal growth conditions were detached (normal condition) and then left in ambient conditions for 9 h (drought). **(B)** Percentage decreases of fresh weight are expressed as percentage water loss. Differences between Bu-5 (WT) and *aod2* were analyzed by Student’s *t*-test (mean ± se, *n* = 3, **p < 0.01, ***p < 0.001).

We examined whether CER10/AOD2 affected the water loss by detaching the whole aerial part of plants and leaving them under ambient conditions. After 9 h under ambient conditions, the *aod2* leaves were much more shriveled than the Bu-5 leaves (Figure 7A). In addition, the rate of water loss from *aod2* leaves was significantly faster than that from Bu-5 leaves (Figure 7B). These findings indicate that CER10/AOD2 plays an important role in water retention of plants.

### Endoplasmic Reticulum Stress in *aod2* Under Osmotic Stress

**Figure 8:**
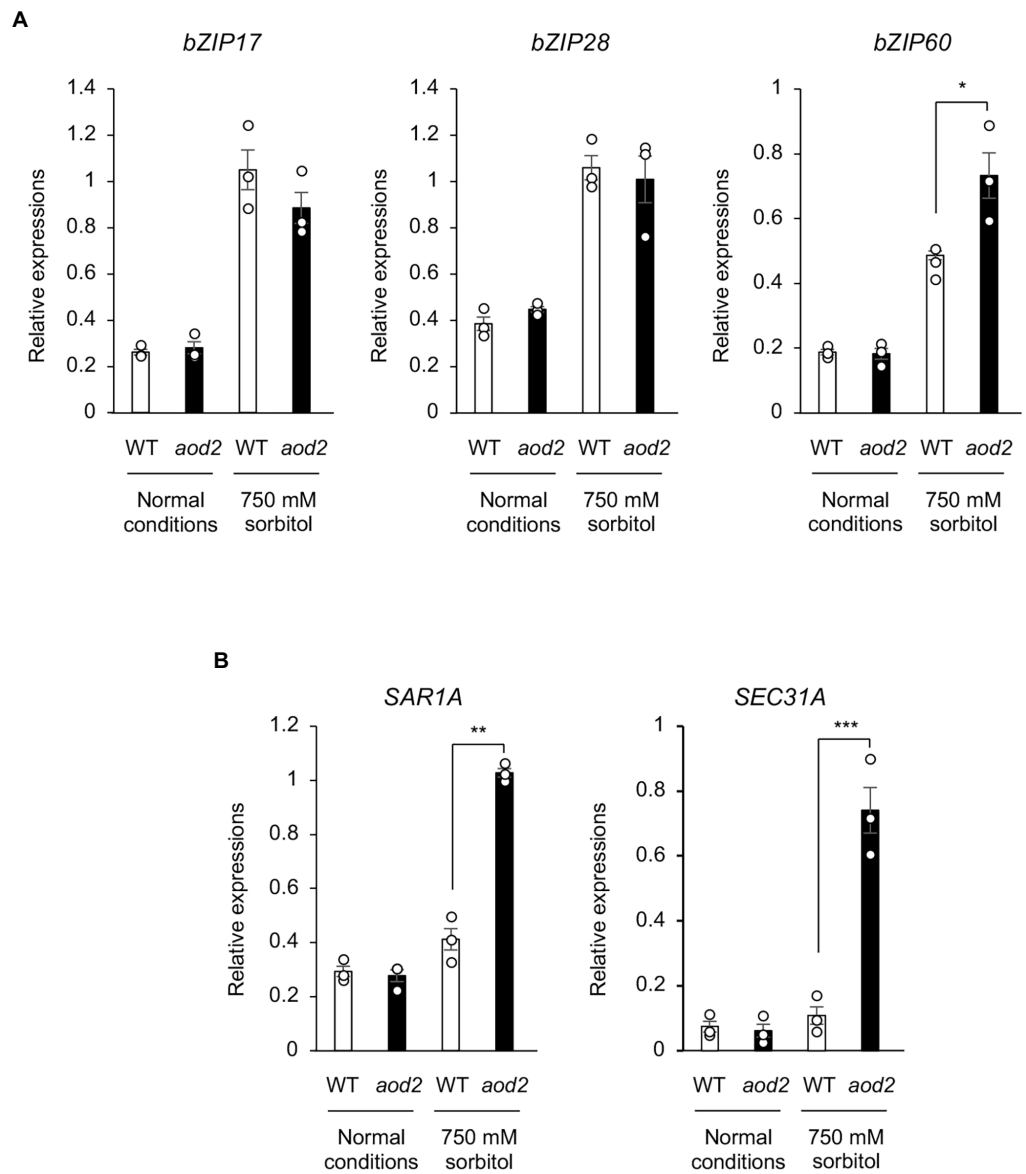
Effect of the *sloh4* mutation on expression of endoplasmic reticulum stress-related genes under osmotic stress. **(A)** Transcript levels of *bZIP17*, *bZIP28*, and *bZIP60* in Bu-5 (WT) and *aod2* plants under normal and acquired osmotic stress (100 mM NaCl for 7 days and subsequent 750 mM sorbitol for 10 days) conditions. **(B)** Expression of the target genes for the transcription factor bZIP60 in WT and *aod2* under normal and osmotic stress conditions; expression levels were determined by quantitative real-time polymerase chain reaction relative to those of *Actin2* (mean ± SE, *n* = 3). Differences between WT and *aod2* were analyzed by Student’s *t*-test. *p < 0.05; **p < 0.01; ***p < 0.001.

In a Col-0-background *cer10* mutant, the loss of *CER10* has been shown to decrease the amounts of membrane sphingolipids and to result in the formation of the Golgi stacks that display aggregate, ring-like structures, suggesting that CER10 plays a role in endocytic membrane trafficking (Zheng et al., 2005). Moreover, defective ER-to-Golgi transport has been shown to impair the salt and long-term heat tolerances in Arabidopsis (Isono et al., 2020). To investigate the differences in the ER stress response in Bu-5 and *aod2* under normal and osmotic stress conditions, we examined the transcript levels of *bZIP17*, *bZIP28*, and *bZIP60*, which are major activators of the ER stress response (Howell, 2013). The transcript level of *bZIP60* in *aod2* was significantly higher than that in Bu-5 under osmotic stress (Figure 8A). Moreover, under osmotic stress conditions, the transcript levels of the two genes regulated by *bZIP60* (i.e., *SAR1A* and *SEC31A*) were also significantly higher in *aod2* than in Bu-5 (Figure 8B).

### Osmotolerance of Mutants Defective in Wax Biosynthesis

**Figure 9:**
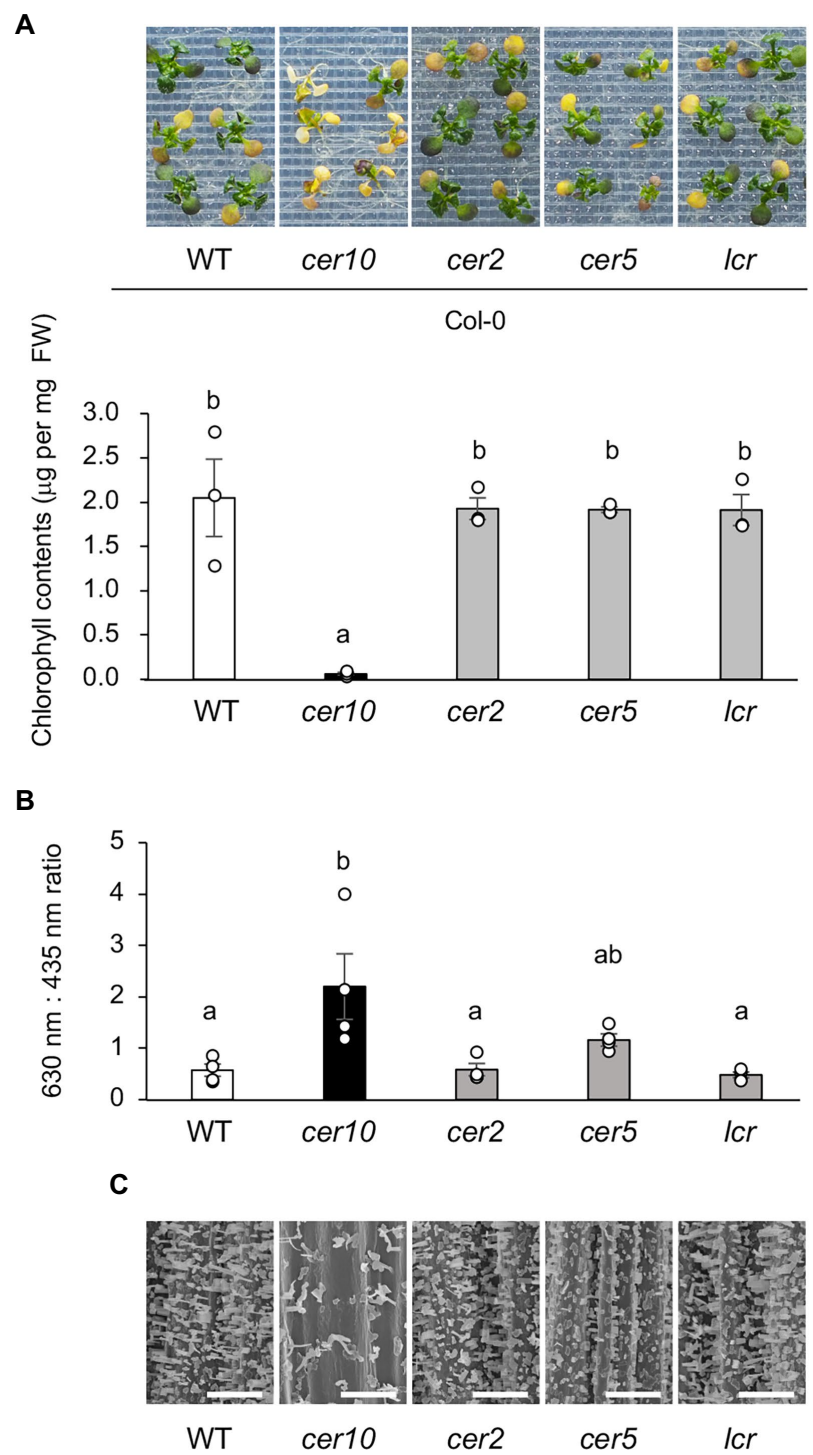
Osmotolerance of mutants defective in wax biosynthesis. **(A)** Top: representative images showing osmo-shock tolerance in Col-0 and Col-0-background *cer10*, *cer2*, *cer5*, and *lcr* mutants. Two-week-old seedlings were mesh-transferred to MS agar plates containing 600 mM sorbitol for 11 days. Bottom: Chlorophyll contents of the mutant plants shown in the top panel. **(B)** Toluidine blue (TB) test using Col-0 and the Col-0-background mutants. TB uptake was examined spectrophotometrically by measuring absorbance at 630 nm (A630). The major peak of absorbance due to plant material (A435) was used for normalization. Relative levels of TB were calculated as the ratio of A630 to A435. **(C)** Scanning electron microscopy images of the surfaces of the stems of Col-0 and the Col-0-background mutants. Bars = 100 μm. Data are presented as mean ± SE, *n* = 3. Letters at the top of columns are based on one-way ANOVA and Tukey’s test, *p* < 0.05.

There has been no comparative study of which genes contributing to cuticular wax load play roles in osmotic tolerance. Therefore, we evaluated the osmotolerance of Col-0 and four Col-0-background mutants defective in cuticular wax load: *cer10*, *cer2*, *cer5*, and *leaf curling responsiveness* (*lcr*; Millar et al., 1999; Pighin et al., 2004; Voisin et al., 2009; Haslam and Kunst, 2013, 2020; Pascal et al., 2013). Of these four mutants, only *cer10* was significantly sensitive to osmotic stress than Col-0 (Figure 9A). To investigate the relationship between the osmotic tolerance of the mutants and wax biosynthesis, the TB-test and observation of epicuticular wax crystals on the stem of the mutants by scanning electron microscopy were conducted. Of the four mutants, *cer10* and *cer5* were stained with significantly more TB compared with Col-0; *cer10* was stained much more than *cer5* (Figure 9B). Consistent with the results of the TB-test, *cer10*, followed by *cer5*, displayed a marked decrease in the number of epicuticular wax crystals on the epidermal surface of the stem compared with that in Col-0 (Figure 9C). Although *cer2* and *cer5* have been reported to show reduced amounts of epidermal wax crystals on the stem surface (Xia et al., 1996; Jenks et al., 2002), we did not observe obvious reductions in the present study. These findings suggest that osmotolerance in Arabidopsis is correlated with wax biosynthesis, and that *CER10* plays a crucial role in both osmotolerance and wax loading in Arabidopsis.

## Discussion

Here, we isolated a new Arabidopsis mutant, *aod2*, showing an osmotolerance-defective phenotype. The causal gene of the phenotype is identical to *CER10*, which encodes an ECR essential for the biosynthesis of VLCFAs. In Arabidopsis, VLCFAs are accumulated in response to osmotic stress, suggesting that VLCFA derivatives, which include components of the cuticular wax and sphingolipids, are an important part of osmotolerance.

It is reported that a defect in *CER10* leads to Golgi aggregation (Zheng et al., 2005). In *aod2*, the expression levels of *bZIP60* and the two genes it regulates (*SAR1A* and *SEC31A*), which are involved in the ER stress response, were increased under osmotic stress compared to the levels in Bu-5. These results suggest that ER stress is enhanced under osmotic stress in *aod2*. It is known that enhancement of ER stress impairs salt and long-term heat stress tolerances in Arabidopsis (Isono et al., 2020). In Arabidopsis, unsaturated aliphatic components account for about 60% of total stem cutin, with C18:2 dioic acid being the predominant unsaturated component (Bonaventure et al., 2004). It is known that FATTY ACID DESATURASE2 (FAD2) affected cutin monomer composition, and the *fad2* mutation causes a 2-fold reduction in the levels of C18:2 dioic acids. The *fad2* mutant exhibited hypersensitivity to tunicamycin, a chemical inducer of ER stress, suggesting that cutin composition and membrane lipid polyunsaturation are involved in ER stress tolerance in Arabidopsis (Nguyen et al., 2019). Very recently, we identified *CYP78A5* encoding a cytochrome P450 monooxygenase known as *KLU* that can confer acquired osmotolerance and heat tolerance to Col-0 WT plants (Kajino et al., 2022). Contrary to the *aod2* mutant, the transgenic Col-0 plants overexpressing *AtKLU* (*AtKLU*ox) developed denser cuticular wax and accumulated higher levels of VLCFAs than WT plants. Furthermore, ER stress induced by osmotic or heat stress was reduced in *AtKLU*ox plants compared to WT plants (Kajino et al., 2022). Taken together, the hypersensitivity of *aod2* mutant to osmotic and long-term heat stresses may be related not only to a decrease in cuticular wax content but also to enhancement of ER stress.

Most intracellular VLCFAs in plant have less than 26 carbon atoms (≤C26), whereas plant cuticular VLCFAs and their derivatives generally have about 30 carbon atoms (Hegebarth et al., 2016). We showed that the major components of the cuticular wax, which include fatty acids, primary alcohols, aldehydes, and alkanes longer than C26, were increased in response to osmotic stress after salt acclimation treatment, suggesting that accumulation of wax components plays an important role in osmotolerance in Arabidopsis Bu-5. Although there was no difference in total wax content between Bu-5 and *aod2*, in the mutant, the amounts of >C30 fatty acids and > C28 primary alcohols and aldehydes were reduced, indicating that these compounds contribute to osmotolerance. The amounts of C26 and C28 fatty acids were notably increased in *aod2* compared with those in Bu-5, suggesting that *CER10/AOD2* plays a role in the biosynthesis of fatty acids longer than C30. Although the amounts of the individual wax components were reduced in *aod2* compared with those in Bu-5, each wax component was still detected, suggesting the presence of other genes possessing ECR activity. Although of the fatty acid elongases, complete loss of KCR1 or PAS2 activity leads to embryonic lethality (Bach et al., 2008; Beaudoin et al., 2009), the loss of *CER10* does not, even though it is the sole ortholog of yeast *TSC13* encoding an ECR (Kohlwein et al., 2001). Screening for revertant mutants that restore acquired osmotolerance in *aod2* or screening for gain-of-function mutants by using approaches such as Full-length cDNA Over-eXpressing gene (FOX) hunting will allow us to identify new genes that encode enzymes with ECR activity (Ichikawa et al., 2006; Higashi et al., 2013; Ariga et al., 2015).

Previously, we reported that Bu-5 was tolerant to salt and osmotic stresses whereas Col-0 was not (Katori et al., 2010). In that study, Bu-5 and Col-0 both showed an increase in wax components under osmotic stress. However, the amounts of >C26 fatty acids and > C28 primary alcohols, C28 and C30 aldehydes, and > C31 alkanes in Col-0 were lower than those in Bu-5. Therefore, the differences in osmotic tolerance between Bu-5 and Col-0 may also be related to the differences in the amounts of these wax components.

When we evaluated the osmotolerance of various mutants lacking in cuticular wax load, we found that the degree of TB staining and the reduction of wax crystals on the epidermal surface were correlated with osmotolerance. However, we also found that the osmotolerance of the *lcr* mutant, which is defective in the amount of cutin but not in the amount of wax (Wellesen et al., 2001), was similar to that of Bu-5. Loss-of-function *myb49* mutants and chimeric *AtMYB49*-*SRDX*-overexpressing *SRDX49* transcriptional repressor plants exhibit decreased amounts of cutin and hypersensitivity to salt stress, suggesting that the amount of cutin correlates with salt tolerance (Zhang et al., 2020). However, *AtMYB49* is involved not only in cutin content but also in the abscisic acid response *via* interaction with ABI5, and *SRDX49* plants show a reduced abscisic acid response (Zhang et al., 2020). Thus, the relationship between reduced cutin content and salt tolerance remains unclear. The osmotolerances of the *cer2* and *cer5* mutants were similar to that of Bu-5. It is reported that the amount of alkanes is reduced in *cer2* compared with that in Col-0, whereas the amounts of fatty acids, aldehydes, and primary alcohols remain unaltered (Pascal et al., 2013). However, we found that there was no difference in the amounts of alkanes in Bu-5, *aod2*, and Col-0, whereas fatty acids, aldehydes, and primary alcohols were decreased in the osmosensitive accessions *aod2* and Col-0 compared with the amounts in the osmotolerant accession Bu-5. These findings suggest that alkanes may not have a significant effect on osmotolerance in Arabidopsis. It has also been reported that the osmotolerance of *cer5* is not affected by the presence of *ABCG11*, a homolog of *CER5* that also contributes to the extracellular transport of wax components (Pighin et al., 2004).

## Conclusion

The Arabidopsis mutant *aod2* lacks *CER10* and shows not only impaired acquired osmotolerance, but also impaired osmo-shock, salt-shock, and long-term heat tolerances. *CER10* encodes an ECR involved in VLCFA biosynthesis. In *aod2*, the amounts of major wax components were decreased, and ER stress mediated by bZIP60 was enhanced under osmotic stress compared with those in Bu-5, indicating that *CER10* plays a crucial role in these abiotic stress tolerances *via* VLCFA metabolism, which is required for cuticular wax synthesis and endocytic membrane trafficking. However, it remains unclear which VLCFA derivatives are critical for osmotolerance. Comparisons between osmotolerance and the amount of VLCFA derivatives in wax load-defective mutants, or screening for revertant mutants in which acquired osmotolerance is restored, should allow us to address this question.

## Data Availability Statement

The datasets presented in this study can be found in online repositories. The names of the repository/repositories and accession number(s) can be found in the article/Supplementary Material.

## Author Contributions

NF and TT initiated, conceived, and coordinated the project. TK isolated the *aod2* mutant. NF identified the causal gene and performed physiological and biochemical analyses. HA and KT performed genome sequencing. NF and TK observed epicuticular wax crystals by scanning electron microscopy with YY. YO determined the wax components. TT wrote the manuscript with assistance from IY and YS. All authors contributed to the article and approved the submitted version.

## Funding

This work was supported by the KAKENHI grants from the Ministry of Education, Culture, Sports, Science and Technology of Japan (19H03092 and 21H05668 to TT) and by the Asahi Glass Foundation, Japan (to TT).

## Conflict of Interest

The authors declare that the research was conducted in the absence of any commercial or financial relationships that could be construed as a potential conflict of interest.

## Publisher’s Note

All claims expressed in this article are solely those of the authors and do not necessarily represent those of their affiliated organizations, or those of the publisher, the editors and the reviewers. Any product that may be evaluated in this article, or claim that may be made by its manufacturer, is not guaranteed or endorsed by the publisher.

## Supplementary Material

Supplementary Figure S1 Oxidative stress tolerance of *acquired osmotolerance-defective* (*aod2*) mutant. Ten-day-old seedlings grown at 22 °C were transferred to Murashige and Skoog agar plates containing 10 μM paraquat for 14 days to induce oxidative stress. Chlorophyll content was determined as an index of the oxidative stress tolerance. Differences between Bu-5 (WT) and *aod2* were analyzed by Student’s *t*-test (mean ± SE, *n* = 3).

Supplementary Figure S2 Polymerase chain reaction-based genotyping of the 439-kbp insertion into the mapped region of the *aod2* genome. Top: Illustration showing the translocation and primer sets (i, ii, and iii) used to detect the insertion. Primer sets (i) and (ii) were designed to detect DNA bands only in the presence of the 439-kbp insertion, and primer set (iii) was designed to detect DNA bands only in the presence of deletion. Bottom: Results of the polymerase chain reaction-based genotyping.

Supplementary Figure S3 Fatty acid (C16–C32) contents in WT and *aod2* plants. Fatty acid (C16–C32) contents of 4-week-old WT and *aod2* plants grown on soil. Differences between WT and *aod2* plants were analyzed by Student’s *t*-test (mean ± SE, *n* = 6, **p* < 0.05, ***p* < 0.01).

Supplementary Figure S4 Wax layer of *aod2*_*At3G55360* plants. Scanning electron microscopy images of the surface of the stems of WT, *aod2*, and *aod2*_*At3G55360*_#3, #4 plants. Bars = 10 μm.

Supplementary Figure S5 Scanning electron microscopy images on leaf surface of *aod2*. Scanning electron microscopy images of the leaf surface between WT and *aod2*. Bars = 50 μm.
