# Supplementary figures and images for: ECERIFERUM 10 Encoding an Enoyl-CoA Reductase Plays a Crucial Role in Osmotolerance and Cuticular Wax Loading in Arabidopsis
Source: Front Plant Sci. 2022 Jun 23;13:898317. doi: 10.3389/fpls.2022.898317 (PMC9259793; doi:10.3389/fpls.2022.898317)

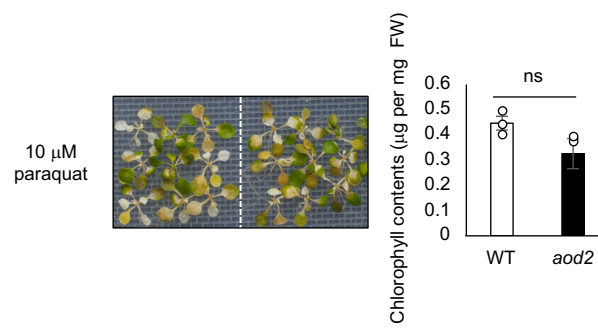

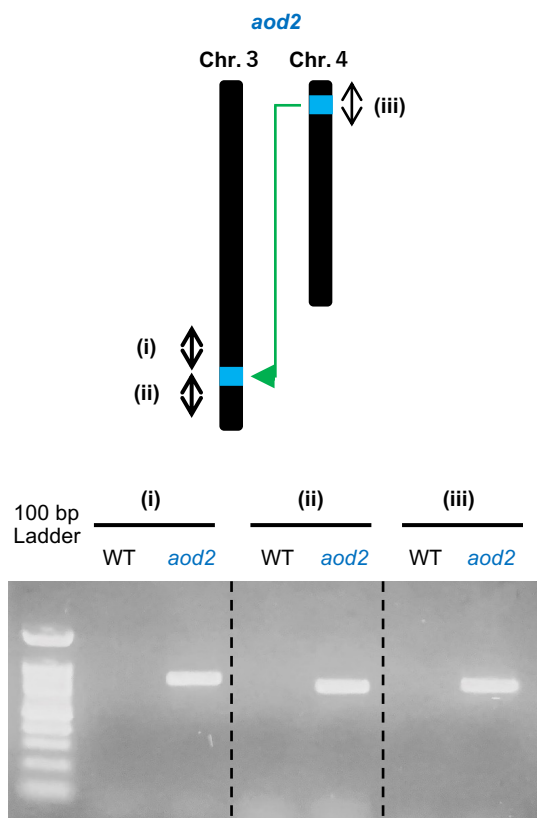

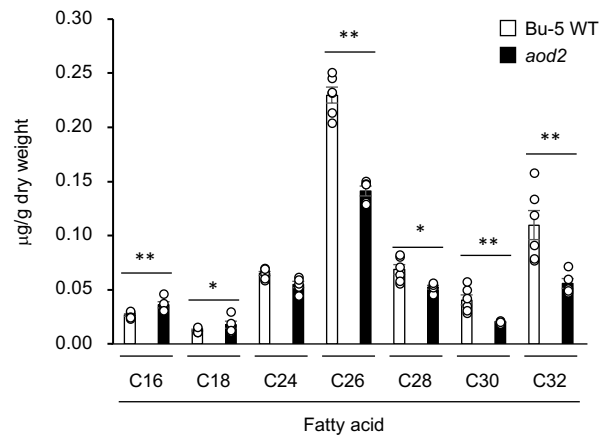

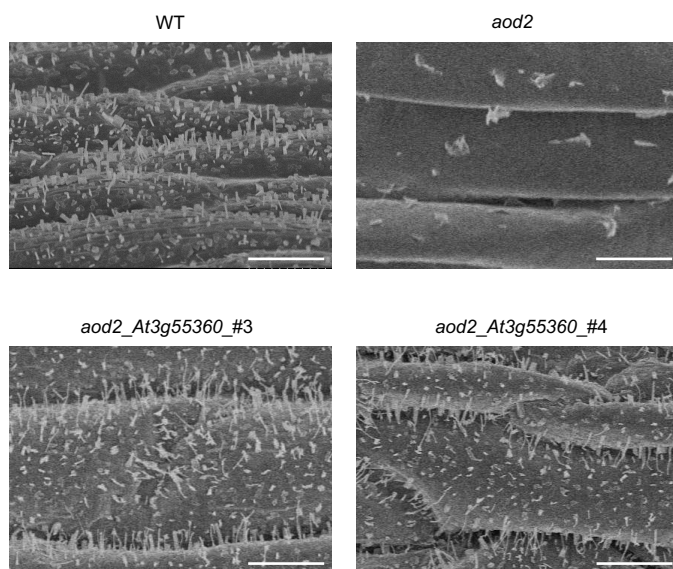

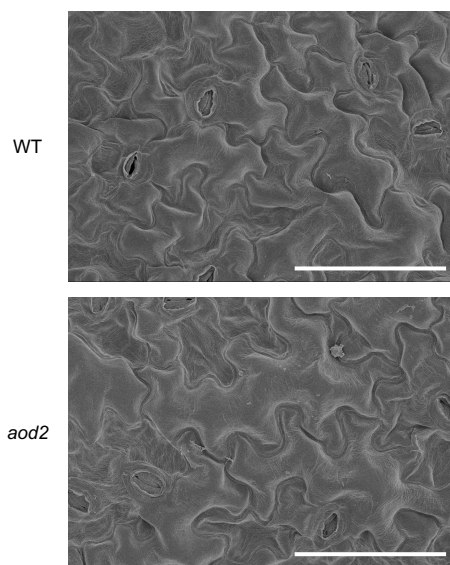

Supplement: Supplementary Figure S1 — Oxidative stress tolerance of acquired osmotolerance-defective (aod2) mutant. Ten-day-old seedlings grown at 22 °C were transferred to Murashige and Skoog agar plates containing 10 μM paraquat for 14 days to induce oxidative stress. Chlorophyll content was determined as an index of the oxidative stress tolerance. Differences between Bu-5 (WT) and aod2 were analyzed by Student’s t-test (mean ± SE, n = 3). [file Data_Sheet_2.PDF]
