# Supplementary material for: ECERIFERUM 10 Encoding an Enoyl-CoA Reductase Plays a Crucial Role in Osmotolerance and Cuticular Wax Loading in Arabidopsis
Source: Front Plant Sci. 2022 Jun 23;13:898317. doi: 10.3389/fpls.2022.898317 (PMC9259793; doi:10.3389/fpls.2022.898317)
Supplement: Supplementary file 2 [file Data_Sheet_1.PDF]

Supplemental Table S1. Primer sets used for quantitative real-time polymerase chain reaction

Primer	Sequence (5′→ 3')
Actin2 qRT F	TCCCTCAGCACATTCCAGCAGAT
Actin2 qRT R	AACGATTCCTGGACCTGCCTCATC
RAB18 qRT F	CCGTTAAGCTTCGAACAATCGTGT
RAB18 qRT R	CAACACACATCGCAGGACGTACA
RD29A qRT F	TGGATCTGAAGAACGAATCTGATATC
RD29A qRT R	GGTCTTCCCTTCGCCAGAA
KIN1 qRT F	GGAAGGCATTCTTGTTGGTCTCTG
KIN1 qRT F	GCCCACATCTCTTCTCATCATCAC
COR15A qRT F	AGCTTCGGCGCTGTCAGAG
COR15A qRT R	GTCACCTTTAGCGGCGTAGA
bZIP17 qRT F	CGTGAAGGTGTTGCAGGTC
bZIP17 qRT R	ACGTTGGTAGCTGCAGGAAT
bZIP28 qRT F	GGAGACTCCAGTGGCGAAT
bZIP28 qRT R	GCGACTGATTTATCATCGGACT
bZIP60 qRT F	CGATGATGCTGTGGCTAAAA
bZIP60 qRT R	TCTCAAGCATTCTCTTTCGAGAT
SAR1A qRT F	TTCACATGCTCAAAGATGAGAGA
SAR1A qRT R	TGCCAATGCTAAGTTCCTCAG
SEC31A qRT F	GATTTCTCTGTATGCAGAACCTGA
SEC31A qRT R	GATTTCTCTTGATATGGATTGGAAA
CER10 qRT F	AGCACAGCCGTCTGAGAAA
CER10 qRT R	GCAACAATGGCTTCTAAAGGA

Supplemental Table S2. Primer sets for simple sequence length polymorphism markers used in this study

Chromosome	Primer	Sequence (5′ → 3′)
Chr. 1	JV26/27 F	CAAGAGATTGCAACATCCACA
	JV26/27 R	AAGCTCCTTGGATCCGATTT
	SO392 F	GTTGATCGCAGCTTGATAAGC
	SO392 R	TTTGGAGTTAGACACGGATCTG
	CIW1 F	ACATTTTCTCAATCCTTACTC
	CIW1 R	GAGAGCTTCTTTATTTGTGAT
	NGA111 F	TGTTTTTTAGGACAAATGGCG
	NGA111 R	CTCCAGTTGGAAGCTAAAGGG
Chr. 2	PLS5 F	GATGCCTTTCTCCTGGTTG
	PLS5 R	AATATAGCCGTCGTCTTCATCA
	NGA361 F	ACATATCAATATATTAAAGTAGC
	NGA361 R	AAAGAGATGAGAATTTGGAC
	C033 F	CTTATGATAGGGTTTGGTTCC
	C033 R	TGCAATAGCGATATTGGATCG
Chr. 3	CHIB F	ATGAGAAGCTATAATTTTTTCAATA
	CHIB R	CTCATATATACAAAGAACTACTATAC
	F24M12-TGF F	GTTCTCTGCATTCCACACATACTCT
	F24M12-TGF R	CTTGGGTATTCTGAAGAGCATAAAT
	NGA6 F	ATGGAGAAGCTTACACTGATC
	NGA6 R	TGGATTTCTTCCTCTCTTCAC
	At3g_20424280 F	AAGGTATTGTCCACTCTTGTTC
	At3g_20424280 R	AATGGGTTGGAAATTATGCATC
	At3g_20488301 F	GTCGTCAGGGTCCGAACTTG
	At3g_20488301 R	GTCGTTGAGTTGAATACTAAC
	At3g_20540707 F	CACCAAATCCAATAAGTCCATT
	At3g_20540707 R	GGTCCAACAAGTTAGTAACAGAAGT
	At3g_20575005 F	CACATAACTCCCCAGCACAGTAAA
	At3g_20575005 R	CGGAAAGAATTTCAGGGTCTAA
	At3g_20649953 F	CTCCAAGACAAGATCTTCCGTTCT
	At3g_20649953 R	GCTGAAATGGAAGTTCCACAATCA
	At3g_20715665 F	GCTCCTTGCTGTTAATGAATCT
	At3g_20715665 R	CTCCTAACCTTTGCACATACCT
Chr. 4	JV30/31 F	CATTAAAATCACCGCCAAAAA
	JV30/31 R	TTTTGTTACATCGAACCACACA
	CIW6 F	CTCGTAGTGCACTTTCATCA
	CIW6 R	CACATGGTTAGGGAAACAATA
	NGA1139 F	TTTTTCCTTGTGTTGCATTCC
	NGA1139 R	TAGCCGGATGAGTTGGTACC
Chr. 5	NGA106 F	TGCCCCATTTTGTTCTTCTC
	NGA106 R	GTTATGGAGTTTCTAGGGCACG
	SO191 F	CTCCACCAATCATGCAAATG
	SO191 R	TGATGTTGATGGAGATGGTCA
	JV75/76 F	CACAATCAGAGGGGGTTGAT
	JV75/76 R	AAATTTTGGGGGAAATGAAA

Supplemental Table S3. Primers used for cloning and sequencing of *CER10*

Primer	Sequence (5' → 3')	Purpose
pRI909 CER10 F	GGCCAGTGCCAAGCTAGTTCCATGTTTCACTTCTTCTA[illegible]	Complementation test
pRI909 CER10 R	GCAGGCATGCAAGCTTGATGATGTTGTCCTCAA	
aod2_chr3_TL_5'_F	GCACTTAAAGACAGACCAGCT	Check for insertion on Chr. 3 and deletion on Chr.4
aod2_chr3_TL_5'_R	CACCACTCTGGAATGTTACCT	
aod2_chr3_TL_3'_F	GAGTAACTCTATGCATGTGGAGA	
aod2_chr3_TL_3'_R	GCACCATGAAGGTCACCGTCGT	
aod2_chr4_F	GGAGAGTCACGGGTAGGCTA	
aod2_chr4_R	GGACGTGGTGGAATCGGTCCT	
